# Supplementary material for: Cooperation with closely bonded individuals reduces cortisol levels in long-tailed macaques
Source: R Soc Open Sci. 2020 May 13;7(5):191056. doi: 10.1098/rsos.191056 (PMC7277283; doi:10.1098/rsos.191056)
Supplement: Electronic Supplementary Material 1 [file rsos191056supp1.pdf]

SUPPLEMENTARY MATERIAL - 1

# Cooperation with closely bonded individuals reduces cortisol levels in long-tailed macaques

*Martina Stocker, Matthias-Claudio Loretto, Elisabeth H.M. Sterck,*
*Thomas Bugnyar & Jorg J.M. Massen*

E-Mail: martina.stocker@univie.ac.at

## Apparatuses

The dyadic test apparatus consisted of a large wooden base plate (125 cm × 60 cm × 1.2 cm) and a smaller sliding board (100 cm × 28 cm × 1.2 cm) that was attached to the base through rails and could be moved out of reach of the animals. Mounted on that board were two transparent cups that held food rewards (60 cm apart) and two metal loops with a 140 cm long rope threaded through them (60 cm apart). The apparatus for one individual was shorter (base: 43 cm × 65 cm × 1.2 cm), featured only one cup, and the rope was not loose but fixed on the sliding board.

## Training procedure

Each individual had to pass 9 out of 10 trials in two consecutive sessions before advancing to the next training stage where macaques were presented with the dyadic loose string task. All subjects accomplished that within the first two sessions. In the loose string task dyads had to cooperate successfully in a total of 20 trials before both subjects could proceed to the test (for number of trials needed to reach criterion see Tab. S1). Note that these 20 successful trials did not have to be achieved within one training session and that training dyads were not used in test conditions. During the training individuals were rewarded with raisins, peanuts, sunflower seeds, dried corn and peas, whereas in the test sessions we only used peanuts to keep the rewards constant.

## Counterbalanced testing

Social conditions (i.e. cooperation and social control) as well as the degree of affiliation of the partner were counterbalanced over the individuals, i.e. eight subjects were first tested with the partner with which they share a stronger social bond, of which four started with the cooperation condition. The other seven started with the rather neutral partner, of which four started with the cooperation condition. The non-social control was conducted after an individual was tested with one partner in both social conditions. When tested with the second partner six individuals started with the same condition as with which they started with their first partner and eight with the other condition.

**Table S1**. **Subjects & Dyads.** For each individual we listed the trainings partner (incl. trials needed to reach test criterium) and test partners, starting with the partner with which the individual shared a stronger bond.
Individuals that were tested with an additional partner: After the control condition Nu did not go into the testing area together with Op (one of the 4 females with an infant). Therefore, we used Ta as rather neutral partner for Nu, however for Op we could not find an appropriate replacement for Nu. To was supposed to be the rather neutral partner for Ta, but eventually Ta's relationship with To was better than the one with Nu. Su, after being tested with two partners, was used as neutral partner for An. For Ch we could not find a second partner.
Since the hormone analysis of one of Nu's and one of Az' samples did not work and we did not have enough material to repeat it, we tested the respective dyad (Nu-Dn, Az-Mc) again at the end of the study. When we first tested Az with Mc his relationship to her was rather neutral, but when tested again their bond was stronger than before. Note that social bonds are given in proportion and are not symmetric. As Wa did not provide saliva samples, she was not included in the statistical analyses but did act as partner for others.

| Individual characteristics | | | | | Test partner | | | | | Trainings partner | | |
|---|---|---|---|---|---|---|---|---|---|---|---|---|
| ID | Sex | Age | Rank | Carried infant | ID | Sex combi | Infant in dyad | Social bond | Coop. success | ID | Sex combi | Trials needed |
| An | female | 12 | 14 | yes | Bi | ff | yes | 0.10 | 7 | Dn | mf | 26 |
| | | | | | Su | ff | yes | 0.02 | 6 | | | |
| Az | male | 3 | 13 | no | Mc | mf | no | 0.00; 0.10 | 14; 12 | Se | mf | 39 |
| | | | | | Ta | mm | no | 0.08 | 12 | | | |
| Ba | female | 10 | 11 | yes | Wa | ff | yes | 0.13 | 11 | Mc | ff | 32 |
| | | | | | Ka | ff | yes | 0.06 | 12 | | | |
| Bi | female | 4 | 3 | yes | An | ff | yes | 0.15 | 7 | Nu | mf | 37 |
| | | | | | Su | ff | yes | 0.03 | 10 | | | |
| Ch | female | 22 | 12 | no | Mc | ff | no | 0.25 | 9 | Ta | mf | 38 |
| Dn | male | 4 | 16 | no | Nu | mm | no | 0.25; 0.15 | 11; 14 | An | mf | 26 |
| | | | | | Wa | mf | no | 0.08 | 13 | | | |
| Ka | female | 7 | 5 | no | To | mf | no | 0.10 | 11 | Op | ff | 29 |
| | | | | | Ba | ff | yes | 0.05 | 12 | | | |
| Mc | female | 4 | 17 | no | Ch | ff | no | 0.39 | 9 | Ba | ff | 32 |
| | | | | | Az | mf | no | 0.00; 0.06 | 14; 12 | | | |
| Nu | male | 3 | 8 | no | Dn | mm | no | 0.20; 0.15 | 11; 14 | To | mm | 26 |
| | | | | | Op | mf | yes | 0.00 | control | | | |
| | | | | | Ta | mm | no | 0.00 | 14 | | | |
| Op | female | 11 | 6 | yes | Se | ff | no | 0.11 | 13 | Ka | ff | 29 |
| | | | | | Nu | mf | yes | 0.00 | control | | | |
| Se | female | 21 | 18 | no | Op | ff | no | 0.12 | 13 | Az | mf | 39 |
| | | | | | Su | ff | no | 0.06 | 13 | | | |
| Su | female | 20 | 10 | no | Se | ff | no | 0.07 | 13 | Wa | ff | 39 |
| | | | | | Bi | ff | yes | 0.04 | 10 | | | |
| | | | | | An | ff | yes | 0.04 | 6 | | | |
| Ta | male | 5 | 7 | no | Az | mm | no | 0.13 | 12 | Ch | mf | 38 |
| | | | | | To | mm | no | 0.13 | 15 | | | |
| | | | | | Nu | mm | no | 0.00 | 14 | | | |
| To | male | 3 | 4 | no | Ka | mf | no | 0.19 | 11 | Nu | mm | 26 |
| | | | | | Ta | mm | no | 0.10 | 15 | | | |
| Wa | female | 4 | 9 | no | Ba | ff | yes | 0.23 | 11 | Su | ff | 39 |
| | | | | | Dn | mf | no | 0.08 | 13 | | | |

Sex combi. – sex combination; Infant in dyad – one or both individuals had an infant; Coop. success – cooperative success (number of successful trials); trials needed – to reach test criterion.

**Table S2.** Summary of the full models (Q1-3)

| Parameter (level) | Estimate | SE | t value | p-value |
|---|---|---|---|---|
| **Q1 - Cooperative success:** Random effect variance: individual ID: 0.26; partner ID: 0.13 | | | | |
| Intercept | 2.28 | 0.70 | 3.28 | 0.00 |
| Cortisol before | -0.06 | 0.07 | -0.96 | 0.34 |
| Social bond | -2.56 | 2.46 | -1.04 | 0.30 |
| Rank | -0.03 | 0.05 | -0.74 | 0.46 |
| **Q2 a - Change in cortisol during cooperation:** Random effect variance: 0.42 | | | | |
| Intercept | -2.48 | 1.81 | -1.37 | 0.18 |
| Success | -0.02 | 0.12 | -0.19 | 0.85 |
| Social bond | -6.25 | 2.80 | -2.24 | **0.04** |
| Sex (*male*) | 1.37 | 0.66 | 2.07 | 0.06 |
| Rank | 0.19 | 0.06 | 3.44 | **0.01** |
| Infant (*yes*) | 0.83 | 0.79 | 1.05 | 0.32 |
| Maternal kin (*non-kin*) | 0.29 | 0.49 | 0.59 | 0.56 |
| **Q2 b - Changes in cortisol during social control:** Random effect variance: 0.42 | | | | |
| Intercept | -1.40 | 0.93 | -1.51 | 0.15 |
| Social bond | -0.31 | 2.49 | -0.12 | 0.90 |
| Sex (*male*) | 1.09 | 0.66 | 1.65 | 0.12 |
| Rank | 0.09 | 0.06 | 1.47 | 0.17 |
| Infant (*yes*) | 0.73 | 0.75 | 0.98 | 0.34 |
| Maternal kin (*non-kin*) | -0.50 | 0.51 | -0.99 | 0.33 |
| **Q3 - Changes in cortisol across all conditions:** Random effect variance: 0.03 | | | | |
| Intercept | -1.99 | 0.54 | -3.70 | 0.00 |
| Condition (coop) | -0.18 | 0.34 | -0.55 | 0.59 |
| Condition (non-social) | 0.33 | 0.43 | 0.77 | 0.45 |
| Sex (*male*) | 0.96 | 0.38 | 2.56 | **0.03** |
| Rank | 0.12 | 0.04 | 3.42 | **0.01** |
| Infant (*yes*) | 0.63 | 0.44 | 1.44 | 0.18 |

**Table S3.** Subset of top-ranked models (with standardized parameters, Δ AICc < 2) used for model-averaging (if null model is not included).

| Explanatory variables of each model | df | logLik | Δ AICc | weight |
|---|---|---|---|---|
| Q1 - Cooperative success | | | | |
| Social bond | 5 | 18.00 | 0 | 0.43 |
| (Null model) | 4 | 16.33 | 0.40 | 0.35 |
| Cortisol before | 5 | 17.37 | 1.26 | 0.23 |
| Q2 a - Changes in cortisol during cooperation | | | | |
| Rank, social bond | 5 | -44.14 | 0 | 0.46 |
| Rank, social bond, sex | 6 | -42.69 | 0.44 | 0.37 |
| Rank | 4 | -46.59 | 1.87 | 0.18 |
| Q2 b - Changes in cortisol during social control | | | | |
| (Null model) | 3 | -45.85 | 0 | 0.43 |
| Rank | 4 | -45.19 | 1.45 | 0.21 |
| Maternal kin | 4 | -45.32 | 1.72 | 0.18 |
| Sex | 4 | -45.35 | 1.78 | 0.18 |
| Q3 - Changes in cortisol during cooperation, social control and non-social control | | | | |
| Rank, sex | 5 | -109.94 | 0 | 0.44 |
| Rank, sex, infant | 6 | -109.01 | 0.57 | 0.33 |
| Rank | 4 | -111.76 | 1.31 | 0.23 |

df – degrees of freedom; logLik – log-likelihood; Δ AICc – differences of the second order Akaike information criterion between the best model and the other top-ranked models; weight – Akaike weight.

## Additional figure

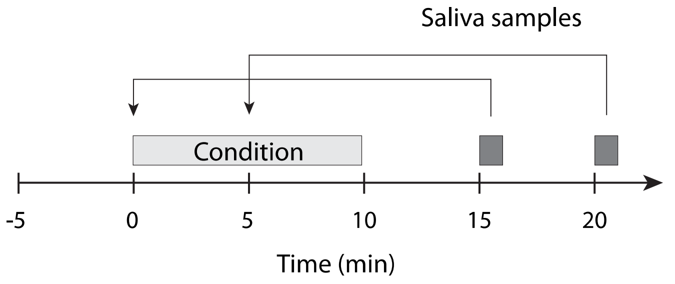


**Figure S1**: Procedure timeline. The macaques had 5 min to habituate to the situation before the test – one of the three conditions (light grey square) – started. After the test, the individuals stayed in the test compartment and gave two saliva samples (dark grey squares), each representing what happened approximately 15 min earlier (arrows).
