# Supplementary material for: Cooperation with closely bonded individuals reduces cortisol levels in long-tailed macaques
Source: R Soc Open Sci. 2020 May 13;7(5):191056. doi: 10.1098/rsos.191056 (PMC7277283; doi:10.1098/rsos.191056)
Supplement: Electronic Supplementary Material 2 [file rsos191056supp2.pdf]

SUPPLEMENTARY MATERIAL - 2

Cooperation with closely bonded individuals reduces cortisol levels in long-tailed macaques

Martina Stocker, Matthias-Claudio Loretto, Elisabeth H.M. Sterck,
Thomas Bugnyar & Jorg J.M. Massen

E-Mail: martina.stocker@univie.ac.at

Salivary cortisol

Biological validation

Prior to the cooperation experiment, we conducted a "stress challenge" to determine how long it takes until a stressor is reflected in salivary cortisol concentrations in long-tailed macaques. Out of four tested individuals (two males, two females), only one very well-trained male (2.5 years old) provided all saliva samples needed.

We observed the male in the group for 30 min before starting the challenge to ensure that at least no obvious stressors occurred. Then we let the subject into the testing compartment and took 13 saliva samples in an interval of 2.5 min, hence for 30 min (approx. 1 min/sample, 1.5 min in-between). In the stress challenge, the experimenter left the room after collection of the first sample. An animal keeper (to the subject unfamiliar) immediately entered the room, stood in front of the subject and made noise with a large paper bag. We chose to present a paper bag because animal keepers have previously observed that this macaque group shows an alert/aversive, though not extremely intense responses to that. After one minute the animal keeper left and the experimenter returned and continued to collect saliva samples.

To determine the influence of the training itself and the presence of the experimenter, we conducted a control test employing the same procedure with the same individuals, but without presenting a stressor. The male was tested in June 2016, first in the control condition, and two days later in the stressor condition, both between 11 and 12 am.

The analysis of the saliva samples collected during the test situations was conducted as described in the *Salivary cortisol – Hormone analysis* section (IBL Cortisol Saliva ELISA, intra-assay CV: 1.56 %).

Results and discussion

The results show a time lag between the start of the stressor and the appearance of the cortisol peak of 15 min (Fig. SC). During that time, the male had a 2.6-fold increase (4.7 ng/ml) in cortisol (3.7-fold (5.6 ng/ml) from the lowest point). During the first half of the control condition the individual showed a highly varying and overall increasing cortisol level (sample 0 – 12.5: mean = 4.13 ± 1.86 SD), which

might be due to interactions in the group or the experimenter starting the session. The level, however, stabilized 12.5 to 15 min after the session has started (sample 15 – 30: mean = 5.72 ng/ml ± 0.42 SD), indicating, first, that the stable cortisol level in the second half represents the stable situation during the control session, and second, that also training and test sessions lead to an elevated level of arousal. In the stress condition the cortisol level finally (after 30 min) drops to the stable, but elevated level shown in the control.

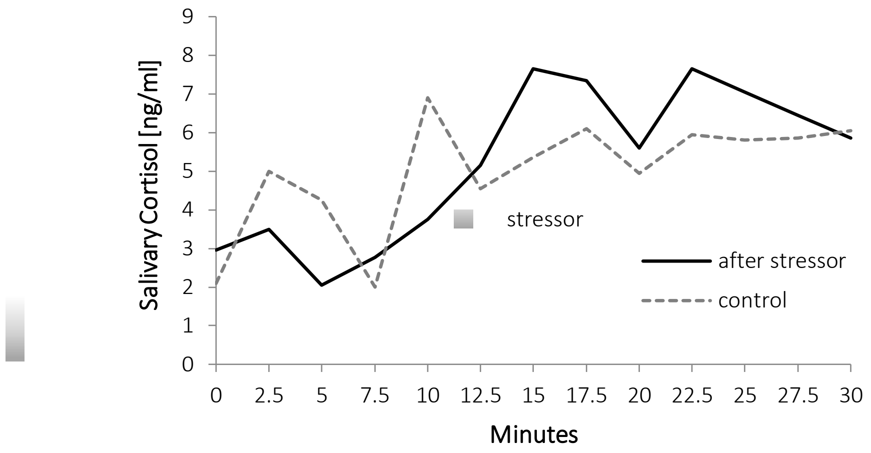

Figure SC. Salivary cortisol of a male long-tailed macaque after a stressor (solid line) and during a control condition (dashed line).

As a precaution regarding animal welfare, we did not keep the monkeys in the training compartment for more than 30 min. Therefore, we were not able to investigate full recovery time in the stress challenge and the second sample during test conditions already had to be taken 10 min after the end of the tests instead of 15 min later. We are aware that one sub-adult male is not necessarily representative for a whole species, but the time lag of 15 min in his salivary cortisol response corresponds with what was shown in blood samples after ACTH challenges in the same species [1,2]. Considering that the peak cortisol concentration in saliva follows the one in blood with a time lag of 2-3 min [3] one might have expected the peak in our male to occur after 17.5 min. However, blood samples after the ACTH challenges were collected only at two time points (probably due to animal welfare concerns) – 15 and 30 min after ACTH administration [1,2], whereas the sampling interval in our stress challenge was much higher, leading to a more detailed temporal resolution. Moreover, the response to ACTH is most likely much more intense than to the rather mild stressor we used in our study.

The male's elevated but stable cortisol level in the stress challenge control indicates that subjects might also experience slightly increased arousal/stress when interacting with an experimenter. However, the mean cortisol level of the second half of this control session (reflecting the subject-experimenter

interaction), which coincides with the overall average level in this study, is somewhat lower than what was found in other studies investigating salivary cortisol in macaques (e.g., long-tailed macaques, *Macaca fascicularis* [4]; rhesus macaques, *M. mulatta* [5]. This suggests that, probably due to regular training with positive reinforcement, most subjects tested in this study did not experience extreme stress during training and test sessions. During the stress test, in which the subject not only showed an endocrine response, but also clear behavioural signs of arousal (piloerection, lip-smacking [1]) that were absent in the control test, salivary cortisol concentrations increased by 4.7 ng/ml. Accordingly, even though changes in cortisol during the stress challenge were rather small, we consider them meaningful.

References

1. Shively CA, Laber-Laird K, Anton RF. 1997 Behavior and physiology of social stress and depression in female cynomolgus monkeys. *Biol. Psychiatry* **41**, 871–882. (doi:10.1016/S0006-3223(96)00185-0)
2. Czoty PW, Gould RW, Nader MA. 2009 Relationship between social rank and cortisol and testosterone concentrations in male cynomolgus monkeys (*Macaca fascicularis*). *J. Neuroendocrinol.* **21**, 68–76. (doi:10.1111/j.1365-2826.2008.01800.x)
3. Lutz CK, Tiefenbacher S, Jorgensen MJ, Meyer JS, Novak MA. 2000 Techniques for collecting saliva from awake, unrestrained, adult monkeys for cortisol assay. *Am. J. Primatol.* **52**, 93–9. (doi:10.1002/1098-2345(200010)52:2<93::AID-AJP3>3.0.CO;2-B)
4. Rapp-Santos KJ, Altamura LA, Norris SL, Lugo-roman LA, Rico PJ, Hofer CC. 2017 Comparison of saliva collection methods for the determination of salivary cortisol levels in rhesus macaques (Macaca mulatta), cynomolgus macaques (Macaca fascicularis), and African green monkeys (Chlorocebus aethiops). *J. Am. Assoc. Lab. Anim. Sci.* **56**, 1–9.
5. Pfefferle D, Plümer S, Burchardt L, Treue S, Gail A. 2018 Assessment of stress responses in rhesus macaques (*Macaca mulatta*) to daily routine procedures in system neuroscience based on salivary cortisol concentrations. *PLoS One* **13**, e0190190. (doi:10.1371/journal.pone.0190190)
